# Strategies of Human T-Cell Leukemia Virus Type 1 for Persistent Infection: Implications for Leukemogenesis of Adult T-Cell Leukemia-Lymphoma

**DOI:** 10.3389/fmicb.2020.00979

**Published:** 2020-05-19

**Authors:** Jun-ichirou Yasunaga

**Affiliations:** Department of Hematology, Rheumatology and Infectious Disease, Faculty of Life Sciences, Graduate School of Medical Sciences, Kumamoto University, Kumamoto, Japan

**Keywords:** human T-cell leukemia virus type 1, Tax, HTLV-1 bZIP factor, adult T-cell leukemia-lymphoma, Foxp3

## Abstract

Human T-cell leukemia virus type 1 (HTLV-1) establishes persistent infection *in vivo* in two distinct ways: *de novo* infection and clonal proliferation of infected cells. Two viral genes, Tax and HTLV-1 bZIP factor (HBZ) play critical roles in viral transcription and promotion of T-cell proliferation, respectively. Tax is a potent transactivator not only for viral transcription but also for many cellular oncogenic pathways, such as the NF-κB pathway. HBZ is a suppressor of viral transcription and has the potential to change the immunophenotype of infected cells, conferring an effector regulatory T cell (eTreg)-like signature (CD4+ CD25+ CCR4+ TIGIT+ Foxp3+) and enhancing the proliferation of this subset. Reports that mice transgenic for either gene develop malignant tumors suggest that both Tax and HBZ are involved in leukemogenesis by HTLV-1. However, the immunogenicity of Tax is very high, and its expression is generally suppressed *in vivo*. Recently, it was found that Tax can be expressed transiently in a small subpopulation of adult T-cell leukemia-lymphoma (ATL) cells and plays a critical role in maintenance of the overall population. HBZ is expressed in almost all infected cells except for the rare Tax-expressing cells, and activates the pathways associated with cell proliferation. These findings indicate that HTLV-1 fine-tunes the expression of viral genes to control the mode of viral propagation. The interplay between Tax and HBZ is the basis of a sophisticated strategy to evade host immune surveillance and increase transmission – and can lead to ATL as a byproduct.

## Introduction

Human T-cell leukemia virus type 1 (HTLV-1) belongs to the delta type retroviruses, which also include bovine leukemia virus (BLV), human T-cell leukemia virus type 2 (HTLV-2) and the simian T-cell leukemia viruses (STLVs) (Hajj et al., 2012). HTLV-1 is a causative agent of several human diseases, including adult T-cell leukemia-lymphoma (ATL), HTLV-1 associated myelopathy (HAM)/tropical spastic paraparesis (TSP), and HTLV-1 uveitis (Matsuoka and Jeang, 2007). ATL is a fatal malignancy of CD4+ T lymphocytes, characterized by pleomorphic leukemic cells with hypersegmented nuclei, called “flower cells,” of a particular immunophenotype similar to regulatory T cells (Tregs) (Karube et al., 2004; Satou et al., 2012).

The HTLV-1 provirus encodes several regulatory and accessory proteins in its pX region. These viral products are involved in viral replication, latency and persistence of infected cells (Franchini et al., 2003). It is thought that the prolonged survival and accelerated proliferation of HTLV-1-infected cells caused by viral factors induces cellular transformation, leading to the onset of ATL after a long latent period. Among the viral genes, *tax* and *HTLV-1 bZIP factor* (*HBZ*), which are encoded in the sense and antisense transcripts, respectively, have been shown to have oncogenic properties in transgenic mouse models (Yasunaga and Matsuoka, 2011). Tax is a pleiotropic viral protein that potently activates viral transcription and dysregulates the transcription and function of many cellular genes. Since Tax is highly immunogenic, its expression is often suppressed *in vivo* (Takeda et al., 2004). On the other hand, transcription of HBZ is consistently detected in all ATL cases (Satou et al., 2006), suggesting that it is critical for the oncogenic mechanism of HTLV-1. The multifarious functions of HBZ have been under study since HBZ was identified in 2002 (Gaudray et al., 2002). It appears that HBZ defines the immunophenotype of HTLV-1-infected and ATL cells, affects the microenvironment and the host immune system, and contributes to persistent infection and pathogenesis. This review summarizes recent findings on the natures and functions of Tax and HBZ and discusses their collaborative role in leukemogenesis by HTLV-1.

## Dynamics of Htlv-1-Infected Cells *In Vivo*

### Modes of Viral Propagation

**FIGURE 1:**
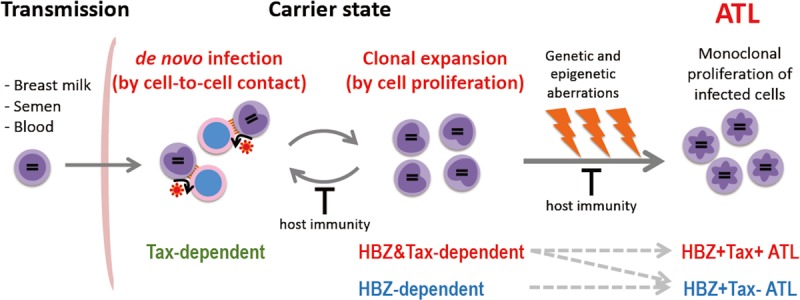
Propagation of HTLV-1-infected cells *in vivo*. HTLV-1 is transmitted by infected cells in breast milk, semen, and blood, and establishes infection by cell-to-cell contact. HTLV-1 spreads *in vivo* by *de novo* infection and clonal expansion. Tax is required for *de novo* infection since Tax drives viral replication. HBZ is critical for clonal expansion. Host immunity controls the number of infected cells and their clonality. In the carrier state, infected cells survive for a long period; genetic/epigenetic aberrations accumulate, and a malignant clone may emerge, resulting in ATL. Approximately half of ATL cases develop in a Tax-independent manner (i.e., Tax– ATL), while the other half retain the capacity to express Tax (Tax+ ATL).

There are two modes of HTLV-1 propagation *in vivo*: *de novo* infection and clonal expansion (Figure 1). *de novo* infection is mediated by infected cells, and is established by integration of the provirus into the host genome. Since HTLV-1 replication is quite low *in vivo*, HTLV-1 transmits from infected cell to uninfected cell through cell-to-cell contact, not via free virions (Igakura et al., 2003; Yasunaga and Matsuoka, 2011). When *de novo* infection occurs, a newly infected cell has a unique integration site of the provirus; thus *de novo* infection increases the variety of HTLV-1-infected clones. On the other hand, clonal expansion is a proliferation of infected cells, which increases the abundance of each clone. It has been reported that HTLV-1-infected clones, in which the provirus is integrated in the same site of the host genome, persisted in infected people over a long period of time (Etoh et al., 1997), indicating that HTLV-1 has the machinery to enhance the proliferation and survival of infected cells. For *de novo* infection, Tax is critical, since it is required for efficient viral replication. In contrast, HBZ appears to play an important role for clonal expansion, since it promotes the proliferation of CD4+ T cells (Satou et al., 2006). Previous studies of seroconverters showed that, early after initial infection, the number of unique clones is high (i.e., the clonality is low) and the proviral load is variable, while both the clonality and the proviral load stabilize within few years (Manns et al., 1999; Okayama et al., 2001; Tanaka et al., 2005). Since Tax is highly immunogenic and *de novo* infection provokes immune activation against HTLV-1, clonal expansion is the dominant way of the virus to persist during the long-term carrier state. The clonal proliferation and prolonged survival of host cells caused by HTLV-1 may also promote cellular transformation, and consequently trigger the onset of ATL.

### Characteristics of Infected Cells

In HTLV-1-infected subjects, the majority of infected cells are CD4+ T cells, and several surface molecules, such as CD25 (IL-2Rα), cell adhesion molecule 1 (CADM1), and C-C chemokine receptor 4 (CCR4), are recognized as markers of HTLV-1-infected cells including ATL cells (Yoshie et al., 2002; Sasaki et al., 2005). However, it is known that HTLV-1 utilizes ubiquitously expressed proteins – glucose transporter GLUT1, heparan sulfate proteoglycan (HSPG), and neuropilin-1 – as receptors for entry into cells (Miyazato and Matsuoka, 2014), and in HTLV-1 carriers the provirus can be detected in not only CD4+ T cells, but also in other lineages of hematopoietic cells: CD8+ T cells, monocytes, B cells, and neutrophils (Koyanagi et al., 1993; Yasunaga et al., 2001; Furuta et al., 2017). A recent study also suggested that hematopoietic stem cells in bone marrow are infected with HTLV-1 and act as a reservoir for infection (Furuta et al., 2017). These facts suggest that HTLV-1 promotes the differentiation of infected cells toward CD4+ T cells with a specific immunophenotype. HTLV-1-infected/ATL cells are known to express several proteins associated with Tregs (Karube et al., 2004; Satou et al., 2012; Sugiyama et al., 2013). Among them, FOXP3 is a master transcription factor of Tregs, and controls the expression of a wide range of genes that exert immune-suppressive functions (Wing et al., 2019). HBZ induces transcription of the *FOXP3* gene, and the number of Tregs in HBZ transgenic mice is significantly increased compared with wild type mice (Satou et al., 2011; Zhao et al., 2011), suggesting that HBZ defines the immunophenotype of infected cells. The detailed functions of HBZ are described later in this review.

## Expression of *Tax* and *HBZ*

### Promoters of *Tax* and *HBZ*

**FIGURE 2:**
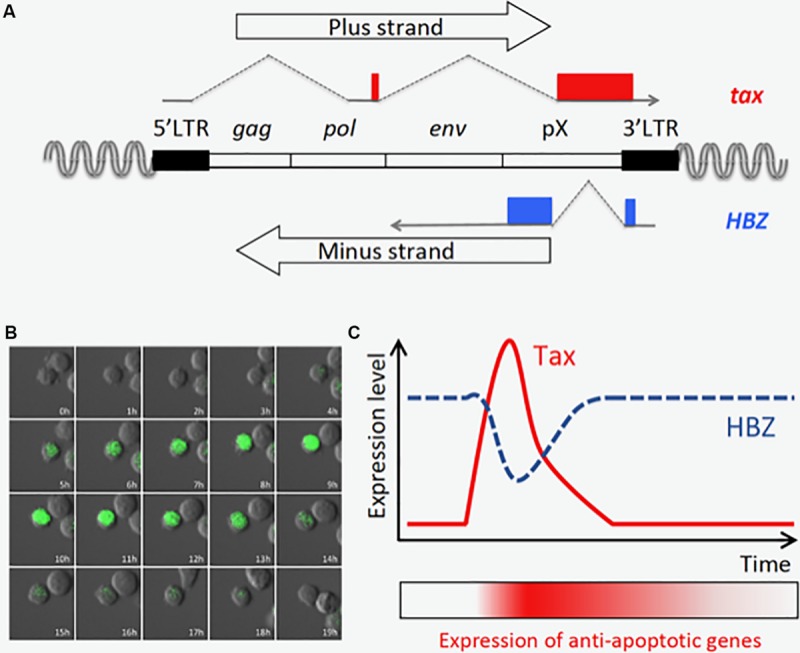
Tax is transiently expressed in an ATL cell line, MT-1. **(A)** Structure of the provirus and the *tax* and *HBZ* genes. **(B)** Time-lapse imaging of Tax expression in MT-1 cells. Destabilized EGFP protein (d2EGFP) expressed under the control of Tax-responsive elements is used to visualize Tax expression. The image was modified from the one obtained in the author’s previous work (Mahgoub et al., 2018). **(C)** Tax is transiently expressed, and HBZ expression is oppositely regulated (Mahgoub et al., 2018). Prolonged induction of anti-apoptotic genes is observed even after Tax is diminished, and this may be important for maintenance of the MT-1 cell population.

The HTLV-1 provirus has two long terminal repeats (LTRs), one at each end, called the 5′LTR and the 3′LTR, respectively (Figure 2A; Matsuoka and Yasunaga, 2013). The 5′LTR acts as a promoter of all transcripts from the plus strand of the provirus (sense transcripts), and several spliced and unspliced viral mRNAs are induced by activation of 5′LTR. Tax is encoded in ORF IV of the doubly spliced sense transcript, and potently activates the 5′LTR through binding to cyclic AMP-responsive element binding protein (CREB) and recruitment of the complex to the Tax-responsive elements (TREs) in the LTR (Kashanchi and Brady, 2005). Since sense transcripts act as the viral genomic RNA and the templates for all structural proteins, such as Gag, Pol, and Env, Tax is critical for viral replication and *de novo* infection to uninfected cells. On the other hand, the minus strand of the provirus encodes two different isoforms of HBZ, spliced (sHBZ) and unspliced HBZ (usHBZ) (Gaudray et al., 2002; Cavanagh et al., 2006; Murata et al., 2006; Satou et al., 2006). It has been reported that *sHBZ* is transcribed from the 3′LTR, and its promoter activity is stronger than that of *usHBZ* (Yoshida et al., 2008). It has been also shown that the sHBZ protein is more abundant than in cells than that of usHBZ (Cavanagh et al., 2006; Murata et al., 2006; Caceres et al., 2018), suggesting that the effect of sHBZ is dominant in infected cells.

### Differences in Expression Patterns Between *Tax* and HBZ

The modes of 5′LTR and 3′LTR activation are quite different from each other; the former is inducible, and the latter is continuous (Mahgoub et al., 2018). Recent studies revealed that several stresses, such as genotoxic stimuli and hypoxic conditions, trigger 5′LTR activation and Tax expression in HTLV-1-infected cells (Kulkarni et al., 2017; Mahgoub et al., 2018). In primary ATL cases, Tax expression is generally suppressed, and approximately half of cases completely lose Tax expression. Three mechanisms for this silencing of Tax are reported: the first is genetic aberration, such as a nonsense mutation or an insertion/deletion in the *tax* gene; the second is deletion of the 5′LTR, and the third is CpG hypermethylation of the 5′LTR (Takeda et al., 2004; Taniguchi et al., 2005). Since Tax is highly immunogenic, silencing Tax expression helps ATL cells evade the host immune system. Importantly, a certain number of infected cells harbor such mutations even in the carrier stage, and these genetic changes seem to occur before proviral integration (Miyazaki et al., 2007; Fan et al., 2010; Katsuya et al., 2019), suggesting that Tax-defective infected cells can survive for a long time and subsequently transform into malignant clones. The other half of ATL cases express low levels of Tax or have the potential to induce Tax. Indeed, it is known that Tax is inducible in half of ATL cases after leukemic cells are transferred to *ex vivo* culture (Kurihara et al., 2005).

In contrast, HBZ is constantly detected, and the sequences of the *HBZ* gene and the 3′LTR are conserved in all ATL cases (Satou et al., 2006; Miyazaki et al., 2007; Fan et al., 2010). There are three SP1 transcription factor binding motifs in the 3′LTR (Yoshida et al., 2008). Since the 3′LTR is epigenetically activated in infected cells (Koiwa et al., 2002; Takeda et al., 2004; Taniguchi et al., 2005; Satou et al., 2016; Miura et al., 2018), and SP1 is ubiquitously expressed in many cell types, it is reasonable to believe that *HBZ* is transcribed in all infected/ATL cells.

Recently, time-lapse imaging showed that Tax is transiently expressed in a small subset (0.5∼3%) of MT-1 cells, a cell line derived from an ATL clone (Figure 2B; Mahgoub et al., 2018). Single cell quantitative RT-PCR also revealed that expression levels of *tax* and *HBZ* are inversely correlated; most MT-1 cells express high levels of HBZ but no Tax, while the rare Tax-expressing cells express less HBZ (Mahgoub et al., 2018). It is technically difficult to observe the transient expression of Tax in primary ATL cells; however, it is reported that fresh ATL cells have similar expression levels of Tax and HBZ compared with MT-1 cells (Usui et al., 2008), suggesting those ATL cases have similar expression profiles of Tax and HBZ *in vivo*. Primary ATL cases can be divided into two subclasses based on Tax status: the Tax-negative type in which Tax is completely silenced, and the Tax-positive type which resembles MT1. Since knockdown of either Tax or HBZ suppresses the proliferation of MT-1 cells (Satou et al., 2006; Mahgoub et al., 2018), both viral factors are thought to be critical for the development of Tax-positive ATL cases.

## Functions of Tax and HBZ

### Tax

Tax is a potent transactivator not only of viral transcription but also of many cellular oncogenic molecules and pathways. It is evident that Tax is an oncoprotein, since Tax can transform primary rodent cells and immortalize human CD4+ T cells, and Tax-transgenic animals develop malignant tumors (Yasunaga and Matsuoka, 2011). Activation of the NF-κB pathway is one of the most important functions of Tax for the survival of infected cells (Matsuoka and Jeang, 2007; Harhaj and Giam, 2018). It was shown that a Tax mutant defective for NF-κB activation could not immortalize T cells (Robek and Ratner, 1999), and transgenic mice containing inducible wild type Tax developed a skin inflammation while mice expressing mutant Tax did not (Kwon et al., 2005). Tax strongly activates the NF-κB pathway through interaction with IKKγ (also known as NEMO), which is a subunit of the IκB kinase (IKK) complex (Sun and Yamaoka, 2005; Currer et al., 2012). Recent biochemical studies revealed that Tax hijacks two E3 ubiquitin ligases, RNF8 and LUBAC, and induces the active macromolecular IKK complex via a process mediated by hybrid polyubiquitin chains (Ho et al., 2015; Shibata et al., 2017).

On the other hand, constitutive Tax expression induces hyperactivation of NF-κB and triggers DNA damage and cellular senescence (Kinjo et al., 2010; Zhi et al., 2011). Therefore, it is disadvantageous for infected/ATL cells to express Tax, although Tax is important for *de novo* infection and cell survival. There must be some way for infected cells to evade Tax-induced cell toxicity and host immune reaction. It was shown that MT-1 cells express Tax for very a short time (∼20 h) in a small subpopulation (Mahgoub et al., 2018), suggesting that transient Tax expression minimizes the risk of its harmful effects. Another way to evade senescence induced by Tax involves HBZ. HBZ suppresses the canonical NF-κB pathway through inhibition of p65/RelA (Zhao et al., 2009; Zhi et al., 2011; Wurm et al., 2012). In addition, in the case of ATL cells, the senescence checkpoint may be inactivated by genetic and/or epigenetic host gene aberrations accumulated during the leukemogenic process (Shudofsky and Giam, 2019).

### HBZ

Among the regulatory/accessory genes encoded in HTLV-1, HBZ is the only gene which is conserved and expressed in all ATL cases (Satou et al., 2006; Miyazaki et al., 2007; Fan et al., 2010). The significance of HBZ in HTLV-1-mediated oncogenesis is proven by both *in vitro* and *in vivo* studies. Forced expression of HBZ in CD4+ T cells promotes cell proliferation (Satou et al., 2006), inhibits apoptosis (Tanaka-Nakanishi et al., 2014), impedes DNA damage responses (Vernin et al., 2014; Takiuchi et al., 2017; Rushing et al., 2018), and mediates transformation of primary CD4+ T cells (Kasugai et al., 2016). Inhibition of HBZ suppresses the proliferation of ATL cell lines (Satou et al., 2006; Nakagawa et al., 2018), indicating that HBZ is critical for the proliferation and maintenance of ATL cells. HBZ transgenic (HBZ-Tg) mice that express HBZ in CD4+ T cells develop systemic inflammation, such as dermatitis and pulmonary alveolitis, and T-cell lymphoma (Satou et al., 2011). In HBZ-Tg mice, production of the immunosuppressive cytokine IL-10 is higher than in wild-type littermates, suggesting that HBZ contributes to the suppression of host immunity (Yasuma et al., 2016). Another transgenic mouse strain that expresses HBZ under the control of the *granzyme B* promoter demonstrates a lymphoproliferative disease and hypercalcemia, which is often seen in ATL patients (Esser et al., 2017). HBZ dysregulates the transcription of many cellular genes using its two different molecular forms, HBZ protein and *HBZ* RNA (Satou et al., 2006; Mitobe et al., 2015). HBZ protein has a bZIP domain in the C-terminus, and forms heterodimers with other host bZIP proteins, such as JUN and CREB/ATF (Ma et al., 2016). In addition, the N-terminus of the HBZ protein contains two LXXLL motifs that are important for its interaction with CBP/p300 (Clerc et al., 2008). Binding of HBZ to these transcription factors and histone modifiers modulates the transcription of cellular and viral genes. HBZ protein targets the Rb/E2F-1 complex and activates the transcription of E2F target genes associated with cell cycle progression and apoptosis (Kawatsuki et al., 2016). It has been shown that HBZ RNA activates the promoters of several host genes, although the precise mechanism is not yet clear (Mitobe et al., 2015). A recent study revealed that HBZ induces a bZIP protein, BATF3, and the BATF3/IRF4 complex promotes the growth of ATL cells by induction of its downstream targets, such as *MYC* (Nakagawa et al., 2018).

Importantly, HBZ induces the transcription of *FOXP3* through the recruitment of Smad proteins and p300 to its promoter region (Satou et al., 2011; Zhao et al., 2011). Since *FOXP3* is the master gene of Treg cells, HBZ-expressing cells have an immunophenotype similar to that of Treg cells. CD4+ T cells in HBZ-Tg mice expresses several surface markers of Tregs, such as CD25, cytotoxic T-lymphocyte associated protein 4 (CTLA-4), glucocorticoid-induced TNF-related protein (GITR), CCR4, and T cell immunoreceptor with Ig and ITIM domains (TIGIT) (Satou et al., 2011; Sugata et al., 2016; Yasuma et al., 2016). CCR4 and TIGIT are innately expressed in the subset of Tregs known as effector type Tregs (eTregs), which have strong suppressive function (Sugiyama et al., 2013; Joller et al., 2014). Although HBZ-expressing cells imitate the Treg phenotype, their regulatory function is impaired, since HBZ suppresses the function of FOXP3 via physical interaction (Satou et al., 2011). In Foxp3-expressing T cells in HBZ-Tg mice, the expression level of several Foxp3 target molecules, such as CD25 and CTLA-4, is lower than Treg cells in WT mice (Satou et al., 2011), indicating that dysfunctional Treg cells are induced by HBZ. In addition, those Treg-like cells of HBZ-Tg mice readily lose Foxp3 expression and produce an excessive amount of IFN-γ, which plays important roles in inflammation and lymphomagenesis induced by HBZ (Yamamoto-Taguchi et al., 2013; Mitagami et al., 2015). HBZ represses inhibitory signaling through TIGIT and PD-1, and keeps cells proliferative in response to activation of the T-cell receptor (Kinosada et al., 2017). Taken together, these observations show that HBZ promotes the differentiation of infected cells into eTreg-like CD4+ T cells that are functionally impaired. Indeed, the immunological phenotype of primary ATL cells resembles that of HBZ-Tg CD4+ T cells, and shares certain characteristics of normal Tregs, whereas the regulatory function is often lost (Karube et al., 2004; Chen et al., 2006; Satou et al., 2012). These findings suggest that HBZ fixes the immunophenotype of HTLV-1-infected cells and may accelerate oncogenic changes in this subset by growth promotion.

## Distinct Roles of Tax and HBZ in Viral Persistence and Pathogenesis

As described above, Tax is transiently expressed in only a tiny fraction of MT-1 cells (Mahgoub et al., 2018). In contrast, the expression of *HBZ* is almost constant; and interestingly, it is contrary to that of *tax* in Tax-expressing MT-1 cells (Mahgoub et al., 2018). Another study, which detected those viral transcripts in HTLV-infected cell lines by RNA-FISH, also demonstrated that *tax* mRNA is highly expressed in cells with low *HBZ* expression (Billman et al., 2017). Since both Tax and HBZ are required for survival of MT-1 cells (Satou et al., 2006; Mahgoub et al., 2018), these two viral products must have collaborative roles in ATL cells. At a single-cell level, Tax-expressing cells express high levels of several anti-apoptotic and NF-κB-related genes, and Tax-negative cells are divided into two subpopulations which expressed medium or low levels of the anti-apoptotic genes. According to these experimental findings and computational simulations, it was hypothesized that transient Tax expression confers an anti-apoptotic property on the expressing cell, and that this effect lasts after Tax expression is diminished (Figure 2C). In contrast, the transcriptome profile in cells that express HBZ dominantly represents activation of the pathways associated with cell proliferation. Hence, in MT-1 cells, the alternating expression of Tax and HBZ seems to execute cooperating programs for survival and proliferation, respectively. Importantly, Tax is induced by various cytotoxic stresses, suggesting that it protects infected cells from apoptosis and triggers viral replication to increase the chance of transmission in a critical situation. The expression patterns and functions of Tax and HBZ comprise a clever strategy of HTLV-1 for persistent infection – a strategy that can also lead to ATL.

## Concluding Remarks

Human T-cell leukemia virus type 1 was first discovered in 1980, and intensive studies of viral replication and cellular transformation have revealed the molecular mechanisms of HTLV-1-induced pathogenesis. Viral proteins, especially Tax and HBZ, are attractive therapeutic targets since the former is sensitive to host immunity and the latter is constantly expressed in ATL cells. Further studies are needed to develop new treatments and prophylactic strategies based on the growing knowledge of HTLV-1 biology.

## Author Contributions

The author confirms being the sole contributor of this work and has approved it for publication.

## Conflict of Interest

The authors declare that the research was conducted in the absence of any commercial or financial relationships that could be construed as a potential conflict of interest.
